# Association of thyroid dysfunction and autoantibody positivity with the risk of preterm birth: a hospital-based cohort study

**DOI:** 10.1186/s12884-022-04806-9

**Published:** 2022-06-08

**Authors:** Jiang-Nan Wu, Ting Peng, Feng Xie, Ming-Qing Li

**Affiliations:** 1 Department of clinical epidemiology, Obstetrics and Gynecology Hospital, Fudan University, 566 Fangxie Rd, Shanghai, 200011 China; 2 Shanghai Key Laboratory of Female Reproductive Endocrine-Related Diseases, Shanghai, China; 3 Department of Obstetrics, Obstetrics and Gynecology Hospital, Fudan University, Shanghai, China; 4 Medical Center of Diagnosis and Treatment for Cervical Disease, Obstetrics and Gynecology Hospital of Fudan University, Shanghai, China

**Keywords:** Thyroid dysfunction, Thyroperoxidas antibody positive, Preterm birth

## Abstract

**Background:**

Evidence for the association of thyroid dysfunction and autoantibody positivity with preterm birth remains controversial. We aimed to study the association of maternal thyroid dysfunction and autoantibody positivity with the risk of preterm birth.

**Method:**

A hospital-based cohort study of 40,214 women was conducted. Gestational age-specific percentiles of the FT4 and TSH concentrations were used for the definition of thyroid dysfunction. Autoantibody positivity was identified when the concentration > the threshold. The association of thyroid dysfunction and autoantibody positivity with the risk of preterm birth was estimated.

**Results:**

No significant higher risk of preterm birth was found for women with variants of thyroid dysfunction or autoantibody positive than euthyroid women. Sensitivity and stratification analyses indicated that thyroperoxidase antibody (TPOAb) positivity in the first trimester (odds ratio [OR], 1.49; 95% confidence interval [CI], 1.17–1.90) and overt hypothyroidism restricted to women negative for TPOAb (OR, 4.94; 95%CI: 1.64–14.84) was associated with an increased risk of preterm birth. Modification effects of gestational age were found for women who had the test ≤18 and > 18 weeks. Continuous FT4 measurements tested ≤18 weeks of gestation were associated with a higher risk of preterm birth (OR, 1.13, 95% CI: 1.00–1.28), while a negative relationship for FT4 concentrations tested > 18 weeks of gestation (OR = 0.68, 95% CI: 0.48–0.97).

**Conclusions:**

Some specific thyroid function abnormalities were associated with an increased risk of preterm birth. Interaction between gestational age and FT4 concentration on the risk of preterm birth was identified, with a critical node of 18 weeks of gestation.

**Supplementary Information:**

The online version contains supplementary material available at 10.1186/s12884-022-04806-9.

## Background

Preterm birth is the leading cause of morbidity and mortality in children < 5 years [1, 2] and is associated with risk of health problems later in life [3]. However, the identified risk factors for preterm birth are largely absent [1, 4].

Maternal thyroid hormone is essential for placentation and fetal development [5, 6]. Gestational thyroid dysfunction is then associated with the risk of adverse fetal outcomes, such as fetal growth restriction and brain underdevelopment [7, 8]. Despite relationships that are linked between overt hypothyroidism and specific hyperthyroidism (e.g., Graves disease) with the risk of preterm birth [5, 9], evidence for the association of milder thyroid dysfunction and autoantibody positivity with preterm birth remains controversial [4, 10–13].

Previous studies in estimating these associations had certain limitations since a lack of a unified definition for thyroid dysfunction and a low statistical power [4]. Recently, a meta-analysis of large individual participant data (47,045 pregnant women) concluded positive associations between mild thyroid dysfunctions and preterm birth [4]. For example, the odds ratio (OR) was 1.29 (95% confidence interval [CI]: 1.01–1.64) for preterm birth among women with subclinical hypothyroidism relative to euthyroid women [4]. However, the heterogeneity of the studies included in the meta-analysis might affect the robustness of the results and produce different findings.

Identification of the association of thyroid dysfunction and autoantibody positivity with the risk of preterm birth and whether these associations have specific characteristics (e.g., gestational age or population-specific) are crucial for guiding the necessity and specificity of clinical intervention for women with disorders. Thus, we conducted a cohort study to address these concerns.

## Methods

### Study design and data collection

A hospital-based cohort study of pregnant women who received prenatal examinations between April 2012 and August 2016 was conducted in 2018 [14, 15]. All pregnant women who visited during the period were included in the cohort. At the first visit, maternal characteristics, such as maternal age, resident location, parity, and conception model, were investigated. Thyroid function was recommended at recruitment. They were then followed up until the 42nd day after the birth. During this period, pregnancy complications (e.g., gestational diabetes mellitus and preeclampsia) and outcomes (e.g., fetal gender, gestational weeks at delivery, weight, and congenital malformations) were identified [14, 15]. Inclusion criterions of subjects included for the analysis in the present study referred to women with singleton pregnancy, who received at least a thyroid function test, and those with confirmed birth outcome. Exclusion criterion referred to pregnant women with multiple pregnancies, without a thyroid function test, and those lost to follow-up. Basic characteristics between women included for analysis and those who were lost to follow-up were imbalanced [15]. Because treatment information was not collected for women with gestational thyroid diseases (e.g., subclinical hypothyroidism, overt hypothyroidism, hyperthyroidism, thyroiditis, thyroid nodules) in the present study, these women were further excluded from the analysis since some drugs (e.g., antithyroid drug) might affect the association between thyroid dysfunction and risk of preterm birth [16]. The reference ranges for the TSH concentration (uIU/mL) in the hospital were 0.05–5.17, 0.39–5.22, and 0.60–6.84 for women who had the test between 1 and 12, 13–28, and 29–40 weeks of gestation; while 12.91–22.35, 9.81–17.26, and 9.12–15.71 for the FT4 concentration (pmol/L), respectively.

### Outcomes

The primary outcome was preterm birth, which was defined as infants born before 37 weeks of gestation. Secondary outcome was very preterm birth (e.g., < 32 weeks of gestation).

### Thyroid function test

Thyroid function, including production of TSH, free and total triiodothyronine (FT3, TT3), FT4, TT4, thyroperoxidase (TPO), TSH receptor (TR), and thyroglobulin (Tg) antibodies were measured with an electrochemiluminescence immunoassay (Roche Elecsys, Germany) at the first visit to the hospital (range from 4 to 39 weeks, with a median of 15.4 weeks of gestation) [14]. The median gestational weeks for the test did not show a significant difference between women with and without a preterm birth baby (*P* = 0.633). Since only 33.5% (13971) of the included women received the test twice and 14.2% (5929) had three measurements. These measurements were not included for analysis (e.g., repeated measurements) in the present study.

Gestational age-specific percentiles of the FT4 and TSH concentrations were used for the definition of thyroid function, including euthyroid (both FT4 and TSH within the range between 2.5th and 97.5th percentile), subclinical hypothyroidism (TSH > 97.5th percentile; 2.5th ≤ FT4 ≤ 97.5th percentile), overt hypothyroidism (TSH > 97.5th percentile; FT4 < 2.5th percentile), isolated hypothyroxinemia (2.5th ≤ TSH ≤ 97.5th percentile, FT4 < 2.5th percentile), isolated hyperthyroxinemia (2.5th ≤ TSH ≤ 97.5th percentile, FT4 > 97.5th percentile), subclinical hyperthyroidism (TSH < 2.5th percentile, 2.5th ≤ FT4 ≤ 97.5th percentile), and hyperthyroidism (TSH < 2.5th percentile, FT4 > 97.5th percentile). TPOAb, TgAb, and TRAb were regarded as positive if the concentrations were > 34 IU/mL, 115 IU/mL, and 1.75 IU/L, respectively [14].

### Covariates

Covariates included maternal age (< 25, 25–34, or ≥ 35 years), residence (local or nonlocal), parity (nulliparous or pluriparous), assisted conception (yes or no), fetal sex (male or female), preeclampsia (yes or no), and gestational diabetes mellitus (yes or no) [14]. Preeclampsia is defined as the onset of hypertension and proteinuria after 20 weeks of gestation [17]. Gestational diabetes was diagnosed based on a 75-g oral glucose tolerance test conducted between the 24th and 28th weeks of gestation [18].

### Data analysis

Prevalence (95% confidence interval, 95% CI) of the outcomes was estimated. Difference between women with and without a preterm birth baby was compared by Chi-squared/Fisher’s exact test (e.g., category variables) or Mann-Whitney U test (e.g., continuous variables). Odds ratio (OR) for the risk of preterm birth among women with thyroid dysfunction was calculated compared with euthyroid women. Adjusted ORs were estimated after controlling for the covariates.

The risk of outcomes in women positive for thyroid antibodies was also assessed relative to those negative for the marker. In these analyses, women without thyroid antibody tests were also included as an unknown antibody status group. Basic characteristics and outcomes (preterm and very preterm birth) were compared among women with and without antibody tests. We conducted sensitivity analyses restricting to those negative for thyroid antibodies. Preeclampsia is an obvious risk factor for preterm birth and women with a TSH concentration > the upper target of the reference might receive potential treatment. Therefore, we further performed sensitivity analyses limited to women without preeclampsia and those with a TSH concentration ≤ the upper targets to exclude these potential impacts. Stratification analyses were performed according to gestation age at the thyroid test (e.g., first, second, or third trimester).

The associations of TSH and FT4 concentrations with the risk of preterm birth were further probed. Outliers (mean ± 4 SD) of the TSH or FT4 concentrations were removed before the analyses. Interaction term analyses were performed to estimate the potential modification of gestational age at the thyroid test on these associations. Stratification analyses were conducted according to the thyroid testing time (e.g., ≤ or > 18 weeks of gestation) because that the fetal thyroid gland is not functionally mature until 18–20 weeks of gestation [19] and that the gestational age specific (12–18 weeks of gestation) association between thyroxine concentration and risk of congenital heart defects was identified in our previous study [15]. Gestational age-specific Z scores or percentiles of the FT4 and TSH were further used in assessing their association with the risk of preterm birth.

All analyses were conducted using Microsoft Office Excel, Stata (version 14.0, Stata Corp., College Station, TX, USA). A two-sided value of *P* < 0.05 was considered statistically significant.

## Results

### Baseline characteristics

**Fig. 1:**
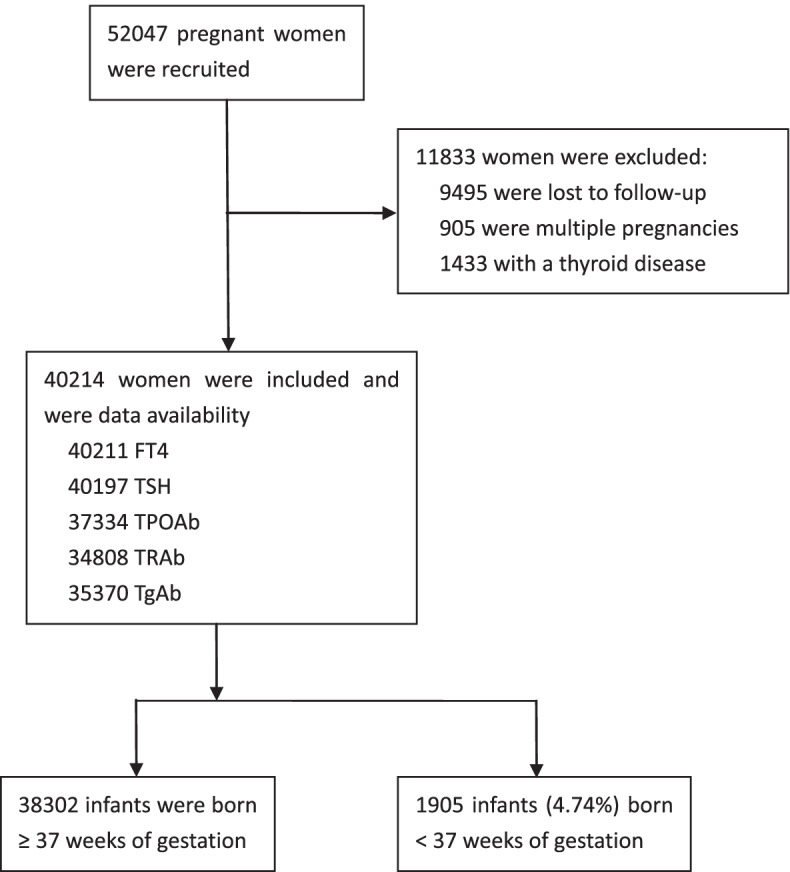
Flow chart of the study

A total of 40,214 (77.3%) women were included in the analysis after excluding women with multiple pregnancies, gestational thyroid disease, and who were lost to follow-up (Fig. 1). Basic characteristics except assisted conception, fetal gender were imbalanced between women with and without antibody tests (Table S1 in the supplementary). Among the included women, 1905 infants were born before 37 weeks of gestation, with a preterm birth rate of 4.74% (95% CI: 4.53 to 5.00) and 0.37% (95% CI: 0.31–0.42) of very preterm birth. Compared with women with a term birth infant, women who delivered a preterm birth baby had a higher positive rate for TPOAb, were older, and were more likely to complicate preeclampsia or gestational diabetes (Table 1).

**Table 1:** Basic characteristics of the study population

Characteristics	Preterm birth		*P* values
	No (*N =* 38,302)	Yes (*N =* 1905)	
Thyroid function			0.082
Euthyroid	34,943 (91.2)	1716 (90.1)	
Subclinical hyperthyoidism	378 (1.0)	27 (1.4)	
Hyperthyroidism	317 (0.8)	18 (0.9)	
Isolated hypothyroxinemia	826 (2.2)	52 (2.7)	
Isolated hyperthyroxinemia	431 (1.1)	21 (1.1)	
Overt hypothyroidism	37 (0.1)	4 (0.2)	
Subclinical hypothyroidism	753 (2.0)	30 (1.6)	
TPOAb positive ^a^	2465 (6.9)	147 (8.2)	0.001
TRAb positive ^a^	683 (1.8)	29 (1.8)	0.121
TgAb positive ^a^	2730 (8.1)	138 (8.1)	0.311
Maternal age, years (median, IQR)	29.0 (27.0–32.0)	29.0 (27.0–32.0)	< 0.001
Gestational week (median, IQR)	39.0 (38.0–40.0)	35.0 (34.0–36.0)	< 0.001
Local residents	29,478 (77.0)	1434 (75.3)	0.088
Nulliparous	32,524 (84.9)	1559 (81.8)	< 0.001
Assisted conception	654 (1.7)	61 (3.2)	< 0.001
Gestational diabetes	3161 (8.3)	196 (10.3)	0.002
Preeclampsia	1986 (5.2)	285 (15.0)	< 0.001
Male fetuses	19,674 (51.4)	1039 (54.5)	0.007

TPOAb, thyroid peroxidase antibody; TRAb, thyroid-stimulating hormone receptor antibody; TgAb, thyroglobulin antibody

^a^ A total of 37,334, 34,808, and 35,370 women were available for TPOAb, TRAb, and TgAb tests

### Association of thyroid dysfunction and antibody positive with risk of preterm birth

**Fig. 2:**
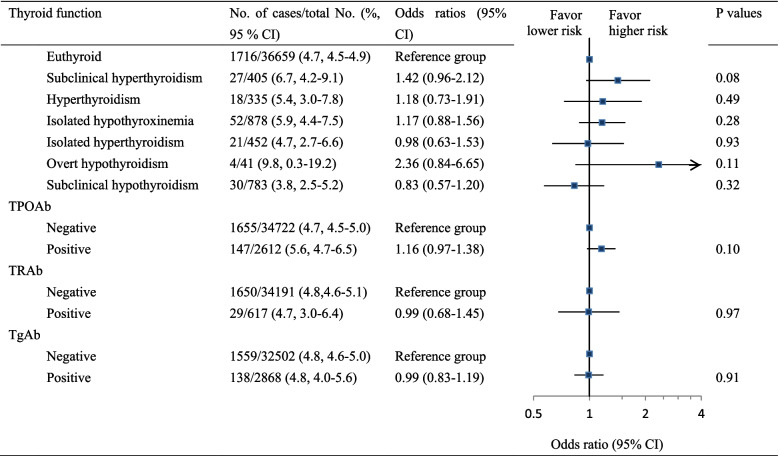
Association of thyroid dysfunction and autoantibody positivity with risk of preterm birth. All analyses were adjusted for maternal age (years), residence (local or nonlocal), parity (nulliparous or pluriparous), assisted conception (yes or no), fetal sex (male or female), gestational diabetes (yes or no), and preeclampsia (yes or no). Thyroid function was classified according to gestational age-specific percentiles of the FT4 and TSH concentrations (the 2.5th and 97.5th percentiles)

Figure 2 shows the association between thyroid dysfunction and the risk of preterm birth. No significant associations were found for variants of thyroid dysfunction. In the sensitivity analysis that we restricted to women negative for TPOAb, overt hypothyroidism was related to a higher risk of preterm birth (OR = 4.94, 95% CI: 1.64–14.84) (Table S2 in the supplementary). Sensitivity analysis restricted to women without preeclampsia and those with a TSH concentration ≤ the upper targets of the reference did not produce significant associations (Table S3 in the supplementary).

Stratification analyses according to the trimesters of the thyroid function test indicated that women with hyperthyroidism diagnosed at the third trimester had a higher but not significant risk to deliver a preterm birth baby compared with euthyroid women (OR, 2.63, 95% CI: 0.91–7.61, *P* = 0.074, Table S3 in supplementary).

Thyroid autoantibody positivity was not significantly associated with preterm birth. However, women positive for TPOAb in the first trimester were significantly related to an increased risk of preterm birth relative to those negative for TPOAb (OR, 1.49, 95% CI: 1.17–1.90, *P* = 0.001) in stratification analyses (Table S4 in the supplementary). There were no significant associations of thyroid dysfunction and antibody positive with the risk of very preterm birth (Table 2).

**Table 2:** Association of thyroid dysfunction and autoantibody positivity with risk of very preterm birth^a^

Thyroid function^b^	β	S.E.	Wald χ^2^	*P* value	OR (95% CI)
Subclinical hyperthyroidism	0.29	0.72	0.16	0.69	1.33 (0.33–5.44)
Hyperthyroidism	−0.11	1.01	0.01	0.91	0.90 (0.12–6.45)
Isolated hypothyroxinemia	0.65	0.39	2.71	0.10	1.91 (0.88–4.12)
Isolated hyperthyroidism	0.24	0.72	0.11	0.74	1.27 (0.31–5.16)
Subclinical hypothyroidism	0.45	0.51	0.79	0.37	1.57 (0.58–4.29)
TPOAb positive (vs. negative)	0.02	0.32	0.01	0.94	1.02 (0.55–1.90)
TRAb positive (vs. negative)	0.73	0.46	2.49	0.11	2.07 (0.84–5.10)
TgAb positive (vs. negative)	0.02	0.30	0.01	0.94	1.02 (0.57–1.85)

^a^ Adjusted factors included maternal age (years), residence (local or nonlocal), parity (nulliparous or pluriparous), assisted conception (yes or no), fetal sex (male or female), gestational diabetes (yes or no) and preeclampsia (yes or no)

^b^ No cases were available for the analysis in women with overt hypothyroidism

### Association of FT4 and TSH concentrations with preterm birth risk

Continuous FT4 concentrations were positive correlated to risk of preterm birth (OR, 1.01, 95% CI: 0.99–1.03), while a negative association was found for continuous TSH measurements (OR, 0.98, 95% CI: 0.94–1.02). However, these associations did not reach statistical significance (*P* = 0.48 and 0.24, respectively). Interaction term analyses showed that there were no modification effects of gestational age on the association of FT4 and TSH concentrations with the risk of preterm birth among the included women.

**Fig. 3:**
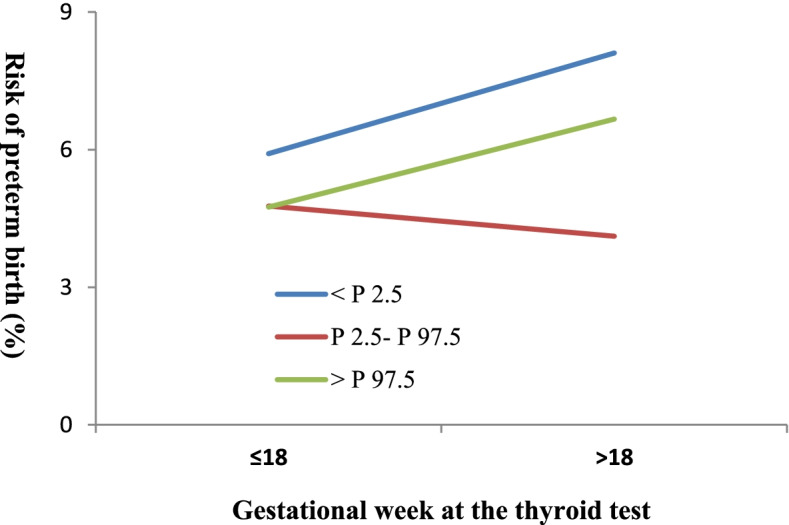
Interaction analyses between gestational age groups (≤ 18 or > 18 weeks) and FT4 concentration categories (< P 2.5, P 2.5 – P97.5, or > P 97.5)

Stratification analyses according to the thyroid function testing time (≤ or > 18 weeks of gestation) did not produce different results for the interaction between TSH and gestational week. Nevertheless, modification effects of gestational age were found for women who had the test ≤18 weeks and > 18 weeks, the corresponding OR for the interaction between FT4 concentrations and gestational age at test was 0.99 (95% CI: 0.98–1.00) and 1.02 (95% CI: 1.00–1.03), respectively (Table 3). Continuous FT4 measurements were associated with a higher risk of preterm birth (OR, 1.13, 95% CI: 1.00–1.28) in women with a test ≤18 weeks of gestation, while a negative relationship for those with a test > 18 weeks of gestation (OR = 0.68, 95% CI: 0.48–0.97). Similar interaction effect was identified in Fig. 3.

**Table 3:** Interaction term analyses between FT4 or TSH concentrations and gestational age at the thyroid function test on risk of preterm birth

Interaction term	Total sample		Women with a test ≤18 weeks of gestation		Women with a test > 18 weeks of gestation	
	OR (95% CI)	*P* value	OR (95% CI)	*P* value	OR (95% CI)	*P* value
FT4 concentrations (pmol/L)	0.98 (0.92–1.04)	0.44	1.13 (1.00–1.28)	0.044	0.68 (0.48–0.97)	0.035
Gestational age at the test (weeks)	0.98 (0.93–1.03)	0.34	1.15 (1.01–1.32)	0.038	0.82 (0.70–0.97)	0.017
FT4 by gestational age at test	1.00 (1.00–1.01)	0.58	0.99 (0.98–1.00)	0.032	1.02 (1.00–1.03)	0.025
TSH concentrations (uIU/mL)	1.04 (0.92–1.18)	0.90	0.97 (0.74–1.25)	0.79	0.95 (0.55–1.64)	0.84
Gestational age at the test (weeks)	1.00 (0.98–1.02)	0.50	1.00 (0.96–1.04)	0.99	0.99 (0.94–1.04)	0.65
TSH by gestational age at test	1.00 (0.99–1.00)	0.26	1.00 (0.98–1.02)	0.89	1.00 (0.98–1.02)	0.90

Adjusted factors included maternal age (years), residence (local or nonlocal), parity (nulliparous or pluriparous), assisted conception (yes or no), fetal sex (male or female), gestational diabetes (yes or no) and preeclampsia (yes or no)

**Fig. 4:**
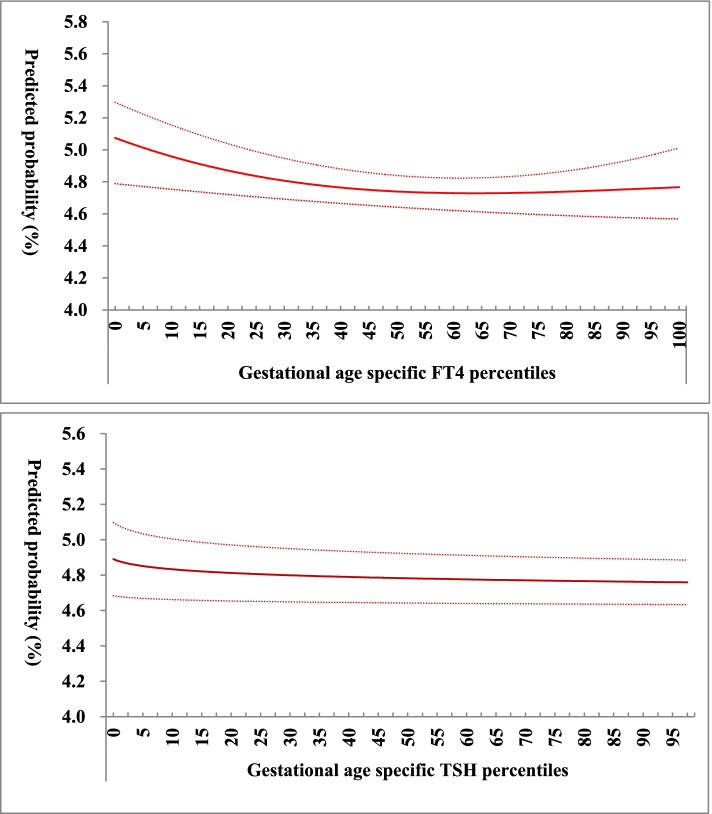
Association of gestational age-specific FT4 and TSH percentiles with predicted preterm birth probability (%, 95 CI). FT4 and TSH concentrations were percentiled by gestational age, higher concentrations ranked lower percentiles

No association between the FT4/TSH concentration Z scores and risk of preterm birth (OR = 1.01 [95% CI: 0.95–1.06] and 0.97 [95% CI: 0.92–1.02], respectively). Figure 4 shows the relationship between the FT4/TSH concentration percentiles and the risk of preterm birth. Women with the lowest percentiles (mean the highest gestational age-specific concentrations) had a higher risk of preterm birth, the risk declined slowly with the increase of percentiles.

## Discussion

The results in the present study did not support that thyroid dysfunction and autoantibody positivity were significantly associated with a higher risk for preterm birth or very preterm birth. However, some specific thyroid function abnormalities, such as TPOAb positive in the first trimester and overt hypothyroidism restricted to women negative for TPOAb, were related to an increased risk of preterm birth. In addition, a modification of gestational age was identified for the association between FT4 concentration and risk of preterm birth, with a critical cut-off value of 18 weeks of gestation.

In comparison with overt hypothyroidism where there is clear evidence for adverse events, the association between mild thyroid dysfunction (such as isolated hypothyroxinemia and subclinical hypothyroidism) and pregnancy outcomes is unclear[20], including preterm birth. It is important to determine whether such thyroid dysfunctions are risk factors for preterm birth since that they are much more frequent than overt thyroid disease [4, 5] and that a clear relationship may provide a basis for possible intervention, such as treatment with levothyroxine.

In the present study, both isolated hypothyroxinemia and subclinical hypothyroidism did not show a significant relation to the risk of preterm birth. These findings were consistent with the pooled results of previous observational studies [12, 13] and might partly explain why levothyroxine treatment did not significantly reduce the risk of preterm birth [21, 22]. However, the finding is different from that in an individual participant meta-analysis [4], in which cohort-specific percentiles were used to define thyroid dysfunction. Pregnancy had a profound impact on the thyroid gland and function [23]. Physiological changes in maternal hormones (e.g., elevated FT4 and decreased TSH) then happen to meet the demands of both the mother and fetus. The change is apparent before 18 ~ 20 weeks of gestation when the thyroid gland of the fetus is not functionally mature [5, 23]. Thus, thyroid dysfunction defined by gestational age-specific percentiles for TSH and FT4 concentrations was more reasonable than the cohort-specific percentile method. Such the method taken in that meta-analysis might induce misclassification bias and then affect the validity of their results [4].

Overt thyroid dysfunction (e.g., overt hypothyroidism or hyperthyroidism) during pregnancy is a known risk factor for preterm birth [5, 13]. The positive correlations between hyperthyroidism and preterm birth in the previous meta-analysis may be mainly contributed to the inclusion of a large Danish population cohort study, which accounts for a 98.7% weight of the pooled OR [13, 24]. An obvious flaw of the definition of hyperthyroidism (based on thyroid function tested before, during, or after pregnancy) raised concerns about a potential classification bias of the disorder and then on its real relation to preterm birth since the prevalence of hyperthyroidism in pregnancy varies depending on the timing of screening [9, 24]. In the present study, we identified the association of overt hypothyroidism with the risk of preterm birth. However, the association was restricted to those negative for TPOAb. Similarly, TPOAb positive only exhibited its impact on preterm birth in the first trimester.

FT4 was deemed a better marker of thyroid function than TSH in pregnancy [5, 25, 26]. In the present study, the FT4 tested ≤ and > 18 weeks of gestation showed different impacts on preterm birth. This disparity might be attributed to different sources of thyroid hormone. Placental transferred maternal FT4 is the only source for fetal demand ≤18 weeks of gestation since the fetal thyroid gland is not functionally mature until 18–20 weeks of gestation. The potential mechanism remains unclear. However, a high FT4 concentration was found to be associated with an increased risk of fetal brain underdevelopment and fetal growth restriction [7, 8]. In our previous study, higher FT4 concentration also showed a relationship with the risk of congenital heart defects and this association became stronger when we restricted to the thyroid measurement tested between 12 and 18 weeks of gestation [15]. Further studies on this disparity effect of FT4 on preterm birth were warranted.

The findings in the present study may partly explain the heterogeneity of the results in the previous studies and might suggest new insights into the potential mechanisms for preterm birth related to thyroid dysfunction [5, 10–13, 27, 28]. Furthermore, these findings may provide evidence for clinical decisions related to a universal thyroid function test and thyroid dysfunction intervention. Clinicians should pay more attention to overt thyroid disease or TPOAb positive at a specific gestational age or in a targeted population. For example, women positive for TPOAb in the first trimester might be the targeted population for the intervention of levothyroxine in reducing the risk of preterm birth. This might explain the failure of levothyroxine in lowing preterm birth risk for women positive for TPOAb before conception in a previous intervention study [29].

### Strengths and limitations

The large sample size in the present study ensured a high statistical power to detect the association between thyroid dysfunction and preterm birth and further probe the specificity of the associations. However, there were some limitations. Firstly, the present study was a single-center study in Shanghai where the iodine status of pregnant women was iodine deficient [30]. In addition, a high loss to follow-up rate (18.2%), imbalanced basic characteristics between women with and with antibody tests, and some important covariates (e.g., maternal BMI and smoking exposure) were not collected in the present study. These disadvantages might result in a potential selection bias and then affect the results. Therefore, multiple-center studies in regions with various iodine statuses for pregnant women are warranted to identify the generalizability of our results. Secondly, thyroid function was measured at different gestational ages rather than a dynamical observation. Thus, we can’t discriminate such specific associations were consequences of hyperthyroidism-persistent effects or results of gestational age-specific functions. Furthermore, treatment information was lacking in the present study. Thus, studies on the impact of dynamic thyroid function measurements and the treatment of gestational thyroid dysfunction on preterm birth were warranted. Finally, the interpretation of causal relationships should be cautious in observational studies.

## Conclusions

In this hospital-based cohort study, women with overt hypothyroidism and negative for TPOAb and those with TPOAb positivity in the first trimester were associated with an increased risk of preterm birth. Interaction between gestational age and FT4 concentration on the risk of preterm birth was identified, with a critical node of 18 weeks of gestation.

## Supplementary Information

**Additional file 1.**

## Data Availability

The datasets generated during and analyzed during the current study are not publicly available due to the provisions of the ethics committee but are available from the corresponding author (wjnhmm@126.com) on reasonable request.
