# Supplementary material for: Association of thyroid dysfunction and autoantibody positivity with the risk of preterm birth: a hospital-based cohort study
Source: BMC Pregnancy Childbirth. 2022 Jun 8;22:473. doi: 10.1186/s12884-022-04806-9 (PMC9175335; doi:10.1186/s12884-022-04806-9)
Supplement: Supplementary file 1 — Additional file 1. [file 12884_2022_4806_MOESM1_ESM.docx]

Table S1. Basic characteristics and outcomes between women with and without antibody tests

|  | Women with a TPOAb test | | | Women with a TRAb test | | | Women with a TgAb test | | |
| --- | --- | --- | --- | --- | --- | --- | --- | --- | --- |
|  | No (N=2873) | Yes (N=37334) | P value | No (N=5399) | Yes (N=34808) | P value | No (N=4837) | Yes (N=35370) | P value |
| Preterm birth (<37 weeks) | 103 (3.6) | 1802 (4.8) | 0.003 | 226 (4.2) | 1679 (4.8) | 0.04 | 208 (4.3) | 1697 (4.8) | 0.13 |
| Very preterm birth (<32 weeks) | 1 (0.03) | 146 (0.39) | 0.002 | 9 (0.2) | 138 (0.4) | 0.009 | 6 (0.1) | 141 (0.4) | 0.003 |
| Local residents | 2381 (82.9) | 28538 (76.4) | <0.001 | 4257 (78.8) | 26662 (76.6) | <0.001 | 3862 (79.8) | 27057 (76.5) | <0.001 |
| Nulliparous | 2500 (87.0) | 31588 (84.6) | <0.001 | 4709 (87.2) | 29379 (84.4) | <0.001 | 4253 (87.9) | 29835 (84.4) | <0.001 |
| Assisted conception | 58 (2.0) | 657 (1.8) | 0.31 | 100 (1.9) | 615 (1.8) | 0.66 | 94 (1.9) | 621 (1.8) | 0.35 |
| Gestational diabetes | 192 (6.7) | 3165 (8.5) | 0.001 | 330 (6.1) | 3027 (8.7) | <0.001 | 260 (5.4) | 3097 (8.8) | <0.001 |
| Preeclampsia | 137 (4.8) | 2136 (5.7) | 0.03 | 257 (4.8) | 2016 (5.8) | 0.002 | 217 (4.5) | 2056 (5.8) | <0.001 |
| Male fetuses | 1479 (51.5) | 19237 (51.5) | 0.97 | 2790 (51.7) | 17926 (51.5) | 0.81 | 2471 (51.1) | 18245 (51.6) | 0.52 |
| Maternal age (median, IQR) | 29 (27-32) | 29 (27-32) | 0.011 | 29 (27-31) | 29 (27-32) | <0.001 | 29 (27-31) | 29 (27-32) | <0.001 |

Table S2. Adjusted odds ratios for preterm births across variants of thyroid dysfunction among women negative for thyroid antibody^*^

| Thyroid function | Women with TPOAb negative | | | | Women with TRAb negative | | | | Women with TgAb negative | | | |
| --- | --- | --- | --- | --- | --- | --- | --- | --- | --- | --- | --- | --- |
|  | No. of women | No. of preterm birth cases (%) | Adjusted odds ratio (95% CI) | P value | No. of women | No. of preterm birth cases (%) | Adjusted odds ratio (95% CI) | P value | No. of women | No. of preterm birth cases (%) | Adjusted odds ratio (95% CI) | P value |
| Euthyroid | 31832 | 1502 (4.7) | 1.00 |  | 31273 | 1502 (4.8) | 1.00 |  | 29846 | 1427 (4.8) | 1.00 |  |
| Subclinical hyperthyroidism | 335 | 22 (6.6) | 1.36 (0.88-2.11) | 0.17 | 314 | 21 (6.7) | 1.36 (0.87-2.14) | 0.18 | 305 | 19 (6.2) | 1.27 (0.79-2.03) | 0.32 |
| Hyperthyroidism | 304 | 18 (5.9) | 1.32 (0.82-2.14) | 0.26 | 294 | 16 (5.4) | 1.18 (0.71-1.97) | 0.52 | 295 | 15 (5.1) | 1.12 (0.66-1.89) | 0.68 |
| Isolated hypothyroxinemia | 730 | 42 (5.8) | 1.11 (0.81-1.53) | 0.52 | 733 | 43 (5.9) | 1.11 (0.81-1.52) | 0.52 | 658 | 38 (5.8) | 1.09 (0.78-1.53) | 0.60 |
| Isolated hyperthyroidism | 376 | 15 (4.0) | 0.81 (0.48-1.37) | 0.44 | 374 | 13 (3.5) | 0.71 (0.40-1.23) | 0.22 | 364 | 14 (3.8) | 0.79 (0.46-1.36) | 0.40 |
| Overt hypothyroidism | **21** | 4 (19.0) | **4.94 (1.64-14.84)** | **0.004** | 38 | 4 (10.5) | 2.51 (0.88-7.11) | 0.08 | 25 | 3 (12.0) | 2.85 (0.84-9.60) | 0.09 |
| Subclinical hypothyroidism | 599 | 22 (3.7) | 0.80 (0.52-1.23) | 0.32 | 660 | 25 (3.8) | 0.81(0.54-1.22) | 0.31 | 556 | 20 (3.6) | 0.77 (0.49-1.21) | 0.25 |

* Adjusted factors included maternal age (years), residence (local or nonlocal), parity (nulliparous or pluriparous), assisted conception (yes or no), fetal sex (male or female), gestational diabetes (yes or no) and preeclampsia (yes or no).

Table S3. Association between thyroid dysfunction and risk of preterm birth among women without preeclampsia and those with a TSH concentration ≤ the upper targets^*^

| Thyroid function | Women without preeclampsia | | | |  | Women with a TSH concentration ≤ the upper targets^#^ | | | |
| --- | --- | --- | --- | --- | --- | --- | --- | --- | --- |
|  | No. of women | No. of preterm birth cases (%) | Adjusted odds ratio (95% CI) | P value |  | No. of women | No. of preterm birth cases (%) | Adjusted odds ratio (95% CI) | P value |
| Euthyroid | 34613 | 1464 (4.2) | 1 |  |  | 36603 | 1715 (4.7) | 1 |  |
| Subclinical hyperthyroidism | 379 | 21 (5.5) | 1.32 (0.85-2.06) | 0.22 |  | 405 | 27 (6.7) | 1.43 (0.97-2.13) | 0.074 |
| Hyperthyroidism | 317 | 15 (4.7) | 1.14 (0.68-1.92) | 0.62 |  | 335 | 18 (5.4) | 1.17 (0.73-1.89) | 0.51 |
| Isolated hypothyroxinemia | 796 | 40 (5.0) | 1.16 (0.84-1.60) | 0.38 |  | 878 | 52 (5.9) | 1.23 (0.93-1.64) | 0.15 |
| Isolated hyperthyroidism | 424 | 18 (4.2) | 1.02 (0.63-1.64) | 0.93 |  | 452 | 21 (4.6) | 1.01 (0.65-1.56) | 0.98 |
| Overt hypothyroidism | 40 | 4 (10.2) | 2.52 (0.90-7.10) | 0.08 |  | 9 | 1 (11.1) | 2.54 (0.32-20.4) | 0.38 |
| Subclinical hypothyroidism | 742 | 24 (3.2) | 0.77 (0.51-1.17) | 0.22 |  | 235 | 8 (3.4) | 0.74 (0.37-1.50) | 0.41 |

* Adjusted factors included maternal age (years), residence (local or nonlocal), parity (nulliparous or pluriparous), assisted conception (yes or no), fetal sex (male or female), and gestational diabetes (yes or no) in the model restricted to women without preeclampsia. Preeclampsia (yes or no) was further controlled in the other sensitivity analysis.

^#^ The upper target for the TSH concentration was 5.17, 5.22, and 6.84 for pregnant women of 1-12, 13-28, and 29-40 weeks of gestation, respectively.

Table S4. Association of thyroid dysfunction and antibody positivity with risk of preterm birth according trimesters at the thyroid function test

| Thyroid function/autoantibody | First trimester | | | | Second trimester | | | | Third trimester | | | |
| --- | --- | --- | --- | --- | --- | --- | --- | --- | --- | --- | --- | --- |
|  | No. of women | No. of preterm birth cases (%) | Adjusted odds ratio (95% CI) | P value | No. of women | No. of preterm birth cases (%) | Adjusted odds ratio (95% CI) | P value | No. of women | No. of preterm birth cases (%) | Adjusted odds ratio (95% CI) | P value |
| Euthyroid | 15006 | 722 (4.8) | 1.00 |  | 19497 | 915 (4.7) | 1.00 |  | 2156 | 79 (3.7) | 1.00 |  |
| Subclinical hyperthyroidism | 104 | 6 (5.8) | 0.99 (0.50-2.78) | 0.66 | 262 | 18 (6.9) | 1.44 (0.88-2.34) | 0.15 | 39 | 3 (7.7) | - |  |
| Hyperthyroidism | 193 | 9 (4.7) | 1.00 (0.62-1.60) | 0.99 | 135 | 9 (6.7) | 1.55 (0.78-3.06) | 0.21 | 7 | 0 (0.0) | 2.63 (0.91-7.61) | 0.07 |
| Isolated hypothyroxinemia | 362 | 19 (5.2) | 0.95 (0.59-1.52) | 0.25 | 472 | 29 (6.1) | 1.21 (0.82-1.78) | 0.34 | 44 | 4 (9.1) | - |  |
| Isolated hyperthyroidism | 118 | 4 (3.4) | 2.27 (0.52-9.90) | 0.27 | 291 | 12 (4.1) | 0.86 (0.48-1.55) | 0.61 | 43 | 5 (11.6) | 1.09 (0.26-4.59) | 0.91 |
| Overt hypothyroidism | 20 | 2 (10.0) | 0.60 (0.31-1.16) | 0.13 | 21 | 2 (9.5) | 2.52 (0.59-10.86) | 0.21 | 0 | - | - |  |
| Subclinical hypothyroidism | 317 | 9 (2.8) | - |  | 416 | 19 (4.6) | 1.01 (0.63-1.61) | 0.97 | 50 | 2 (4.0) | - |  |
| TPOAb negative | 14768 | 686 (4.6) | 1.00 |  | 18997 | 914 (4.8) | 1.00 |  | 447 | 25 (5.6) | 1.00 |  |
| TPOAb positive | 1127 | 78 (6.9) | **1.49 (1.17-1.90)** | **0.001** | 1417 | 66 (4.7) | 0.93 (0.72-1.20) | 0.58 | 27 | 1 (3.7) | 0.65 (0.09-5.02) | 0.68 |
| TRAb negative | 14986 | 717 (4.8) | 1.00 |  | 18287 | 887 (4.9) | 1.00 |  | 432 | 21 (4.9) | 1.00 |  |
| TRAb positive | 270 | 11 (4.1) | 0.88 (0.48-1.63) | 0.69 | 328 | 17 (5.2) | 1.08 (0.66-1.78) | 0.76 | 12 | 1 (8.3) | 1.84 (0.23-15.08) | 0.57 |
| TgAb negative | 14299 | 677 (4.7) | 1.00 |  | 17352 | 838 (4.8) | 1.00 |  | 415 | 22 (5.3) | 1.00 |  |
| TgAb positive | 1370 | 65 (4.7) | 0.97 (0.75-1.26) | 0.83 | 1440 | 68 (4.7) | 0.99 (0.76-1.27) | 0.91 | 19 | 0 (0.0) | - |  |

* Adjusted factors included maternal age (years), residence (local or nonlocal), parity (nulliparous or pluriparous), assisted conception (yes or no), fetal sex (male or female), gestational diabetes (yes or no) and preeclampsia (yes or no).
